# Involvement of a MYB Transcription Factor in Anthocyanin Biosynthesis during Chinese Bayberry (*Morella rubra*) Fruit Ripening

**DOI:** 10.3390/biology12070894

**Published:** 2023-06-21

**Authors:** Saisai Li, Yijuan Zhang, Liyu Shi, Shifeng Cao, Wei Chen, Zhenfeng Yang

**Affiliations:** College of Biological and Environmental Sciences, Zhejiang Wanli University, Ningbo 315100, China; ssli1212@zwu.edu.cn (S.L.);

**Keywords:** Chinese bayberry, anthocyanin, activator, MYB transcription factor

## Abstract

**Simple Summary:**

Color is one of the most significant quality characteristics of Chinese bayberry (*Morella rubra*). Exploration of the anthocyanin biosynthesis mechanism is crucial for the genetic improvement of color quality. Many MYB transcription factors are reported to control the biosynthesis of anthocyanins in a variety of plant species. However, it is still unclear how the *MYB* genes in Chinese bayberry contribute to the synthesis of anthocyanins. In this research, we isolated the MYB transcription factor, MrMYB9, based on comparative transcriptome analysis of red and white Chinese bayberry fruits. We also profiled *MrMYB9* gene expression during the maturation period and in specific tissues. Through bioinformatics and molecular biology experiments, MrMYB9 was subsequently identified as a transcription factor of the R2R3-MYB type associated with anthocyanin biosynthesis. Our research sheds insights on the biosynthesis of anthocyanins during color maturation in the Chinese bayberry.

**Abstract:**

Anthocyanin is a class of water-soluble flavonoids found in Chinese bayberry (*Morella rubra*) that is not only responsible for the variety of colors visible in nature but also has numerous health-promoting benefits in humans. Through comparative transcriptomics, we isolated and identified a transcription factor (TF) of the R2R3-MYB type, MrMYB9, in order to explore the anthocyanin biosynthesis pathway in red and white Chinese bayberries. MrMYB9 transcript was positively correlated with anthocyanin level and anthocyanin biosynthetic gene expression during Chinese bayberry fruit maturation (R-values in the range 0.54–0.84, *p* < 0.05). Sequence analysis revealed that MrMYB9 shared a similar R2R3 domain with MYB activators of anthocyanin biosynthesis in other plants. MrMYB9 substantially transactivated promoters of anthocyanin biosynthesis-related EBGs (*MrCHI*, *MrF3’H*, and *MrANS*) and LBGs (*MrUFGT*) upon co-expression of the *AtEGL3* gene. Our findings indicated that MrMYB9 may positively modulate anthocyanin accumulation in Chinese bayberry.

## 1. Introduction

Due to variations in anthocyanin levels in cultivars, the Chinese bayberry (*Morella rubra*) fruit possesses a wide spectrum of colors, from dark red-purple (Biqi, BQ) to white (Shuijing, SJ), rendering it a fruit crop with high commercial value [1].

Anthocyanins are natural pigments that are extensively produced in plants and are responsible for giving certain plant organs their characteristic red, purple, and blue colors. In addition to attracting pollinators and seed dispersers [2], they also play crucial roles in protecting plants from biotic and abiotic stressors [2,3,4,5]. Anthocyanins are also helpful to human health due to their potent antioxidant and anti-mutant characteristics. Increasing evidence indicates that anthocyanin ingestion lowers the risk of cardiovascular disease, age-related degenerative diseases, and various kinds of cancers [6,7,8].

The anthocyanin biosynthesis pathway has been extensively investigated as a metabolic network rather than a strictly linear pathway. This pathway comprises numerous branches and alternative metabolic routes [9,10]. A variety of enzymes, including chalcone synthase (CHS), chalcone isomerase (CHI), flavanone 3-hydroxylase (F3H), and flavanone 3’-hydroxylase (F3’H) are responsible for the biosynthesis of flavonol, anthocyanin, and proanthocyanidin (PA) [11,12,13,14,15]. Dihydroflavonols are synthesized by these enzymes and are then either transformed to flavonols by flavonol synthase (FLS) or reduced to colorless leucocyanidins by dihydroflavonol 4-reductase (DFR) [16,17,18]. Leucocyanidins are the fundamental structures of flavonoids and anthocyanins. The final stage that both the anthocyanin and PA processes share is promoted by an enzyme called anthocyanin synthase (ANS) [19,20]. UDP-flavonoid glucosyltransferase (UFGT) catalyzes glycosylation, which is the ultimate stage of modification in the anthocyanin pathway [21,22].

The structural genes responsible for anthocyanin biosynthesis are generally governed by three distinct classes of TFs: R2R3-MYB, basic helix-loop-helix (bHLH), and WD40 repeat families [23,24,25,26]. MYB TFs are responsible for the functional specificity of the complex as well as for determining which pathways are regulated [2,27,28]. To date, a large number of MYB TFs controlling anthocyanin biosynthesis have been discovered in many plants. Activators responsible for anthocyanin accumulation covered apple MdMYB10, MdMYB3, MdMYB24L, MdMYB308L, and MdMYB114 [29,30,31,32,33], strawberry FaMYB5 [34], peach PpMYB10.1 and PpMYB6 [35,36], Chinese bayberry MrMYB1 [37], Lily LvMYB5 and LvMYB19 [38,39], *Pistacia chinensis* PcMYB113 [40], and grapevine VvMYBA1 and VvMYBA2 [41,42]. Recent researches revealed that MdNAC42, a novel NAC transcription factor, interacts with MdMYB10 to modulate the anthocyanin level in red-fleshed apples. By increasing and inhibiting the activities of anthocyanin-related genes (*MdF3H*, *MdDFR*, and *MdUFGT*) and sugar-related genes (*MdCWI1*, *MdVGT3*, and *MdTMT2*), the MdMYB305–MdbHLH33–MdMYB10 complex balanced anthocyanin and sugar levels in red-fleshed apples [43,44]. FaMYB5 was also identified as an R2R3-MYB activator that is a component of the FaMYB5–FaEGL3–FaLWD1-like complex, which promotes anthocyanin and proanthocyanidin accumulation by directly targeting the *F3’H* and *LAR* promoters in strawberry [34,45].

In addition, a number of MYB repressors, including strawberry FaMYB1 [46], apple MdMYB16, MdMYB15, and MdMYB306L [47,48,49], banana MaMYB4 [50], peach PpMYB18 [51], grapevine VvMYB4L and VvMYBC2L2 [52,53], Chinese bayberry MrMYB6 [54], Lily LhR3MYB1 and LhR3MYB2 [55], and Petunia PhMYB27 [56], inhibited anthocyanin biosynthesis. Banana MaMYB4 inhibited the expression *of CHS*, *ANS*, and *DFR* genes, leading to decreased anthocyanin production [50]. PpMYB18 negatively regulated the accumulation of anthocyanin and proanthocyanidin in peaches by suppressing the expression of flavonoid-related genes [51]. According to the Ref. [54], MrMYB6 negatively controlled anthocyanin accumulation in Chinese bayberry by forming functional complexes with MrbHLH1 and MrWD40-1 that directly suppressed the activity of *MrANS* and *MrUFGT* gene promoters. However, *MYB* genes, which control the anthocyanin pathway in Chinese bayberry, are still poorly understood. The recent genome sequencing of *Morella rubra* [57] has boosted the likelihood of identifying the MYB gene family associated with anthocyanin biosynthesis in Chinese bayberry.

This study aimed to identify the MYB regulators involved in the anthocyanin pathway in *Morella rubra*. In order to modulate fruit coloration during maturation, the study also investigated how MYB TFs regulate the genes involved in anthocyanin biosynthesis.

## 2. Methods

### 2.1. Plant Materials

Chinese bayberry (*Morella rubra* Sieb. and Zucc.) plants of the ‘Shuijing’ (SJ) and ‘Biqi’ (BQ) varieties, which are grown in the cities of Shaoxing and Cixi, respectively, were harvested for their stems, buds, leaves, and fruits. The fruits were collected 57, 71, 85, 99, and 113 days after full blossom (DAFB).

*Nicotiana benthamiana* L. seeds were planted in containers with a perlite, peat, and vermiculite soil combination and grown for 5–6 weeks in a greenhouse at 25 °C and 55% relative humidity under a 16-h light/8-h dark cycle.

### 2.2. Anthocyanin Content Determination

The anthocyanin concentration was measured using a modified version of the pH-differential procedure [58,59]. The freeze-dried tissue (approximately 1.0 g) was extracted at 4 °C for 12 h using 5 mL of extraction solution (0.3% HCl/methanol). Following 15 min of centrifugation at 10,000× *g*, the resulting product was shifted to a sterile tube and the particulates extracted two or three times with extraction solution until no red color remained in the precipitate. The combined supernatants were diluted to 25 mL. A UV-1750 spectrophotometer (Shimadzu, Japan) was used to measure absorbance at 510 nm and 700 nm in pH 1.0 and pH 4.5 buffers. The anthocyanin concentration was determined in terms of cyanidin-3-O-glucose equivalent using the following equation: TA (mg/100 g) = A × MW × 5 × 100 × 25/e, where TA represents the total quantity of anthocyanin and A = [(A_510_ − A_700_)_pH1.0_ − (A_510_ − A_700_)_pH4.5_]. The molecular weight (MW) was 449.2 and the molar absorption (e) was 26,900. Each biological replicate sample was measured three times.

### 2.3. Extraction of DNA and RNA and Synthesis of First-Strand cDNA

DNA was obtained following the instructions of a FastPure Plant DNA Isolation Mini Kit (Vazyme, Nanjing, China). Total RNA was extracted from Chinese bayberry (*Morella rubra*) using a Plant RNA Extraction Kit (Tianenze, Beijing, China) following the instructions provided by the manufacturer. First-strand cDNA was synthesized from 1 μg of the total RNA using a SuperRT cDNA synthesis kit (Vazyme, Nanjing, Jiangsu, China).

### 2.4. Real-Time Quantitative PCR (RT-qPCR) Analysis

RT-qPCR assays were conducted in triplicate using Yeasen Hieff^®^ qPCR SYBR Green Master Mix (High Rox Plus) on a StepOnePlus^TM^ system (Applied Biosystems, Foster city, CA, USA). The relative expression of each target gene was calculated using the 2^−ΔCt^ method and standardized with the internal reference gene *MrActin* (GQ340770). Supplemental Table S1 details the gene-specific primers used in the experiment.

### 2.5. Analysis of Subcellular Localization

*MrMYB9* cDNA was cloned using RT-PCR and inserted into the pCAMBIA1301-GFP plasmid with specific primers (Supplemental Table S1) and confirmed via DNA sequencing. As previously described [54], the control and recombinant vectors were introduced into *N. benthamiana* leaves via agroinfiltration. Images of GFP fluorescence signals in the FITC (EGFP) channel were captured using a confocal laser microscope system (Nikon A1+, Tokyo, Japan) 60 h after infiltration.

### 2.6. Dual Luciferase Transient Assay

Supplemental Table S1 lists all the gene-specific primers used for the construction of the numerous plasmids in this study. On the basis of the sequences reported [37], the promoter sequences of the anthocyanin biosynthesis-related genes *MrCHI, MrF3′H*, *MrDFR*, *MrANS*, and *MrUFGT* were amplified directly using RT-PCR. Individually obtained promoters were subcloned into the pGreenII 0800-Luc vector. Infiltrations and transient expression analysis were conducted in accordance with previously described protocols [60]. The ratio of firefly and Renilla luciferase transactivation activities was measured based on the instructions supplied by the manufacturer of a Dual-Luciferase Reporter Gene Assay Kit (Yeasen Biotech, Shanghai, China).

### 2.7. Syntenic, Phylogenetic, and Statistical Analysis

The evolutionary relationship between *Morella rubra* MrMYB9 and MYBs of other species, including *Arabidopsis*, walnut (*Juglans regia*), peach (*Prunus persica*), apple (*Malus domestica*), and grape (*Vitis vinifera*) was investigated using synteny analysis. The One Step MCScanX and DualSyntePlot programs were used to analyze the synteny relationship using TBtools [61].

Following alignment using the ClustalW algorithm, phylogenetic trees were generated in MEGA 11.0 using the neighbor-joining approach, with 1000 bootstrap replicates. Pearson’s correlation coefficients were determined to compare the relationship between MrMYB9 transcript pattern, anthocyanin biosynthetic genes, and anthocyanin content in bayberry. SPSS 22.0 software (SPSS Inc., Chicago, IL, United States) was used for statistical analysis. The values are presented as mean ± SD and were analyzed using Duncan’s multiple range test (*p* < 0.05) and Student’s *t*-test (* *p* < 0.1, ** *p* < 0.05, *** *p* < 0.01).

## 3. Results

### 3.1. Anthocyanin Levels in Two Cultivars during the Ripening of Chinese Bayberry

**Figure 1:**
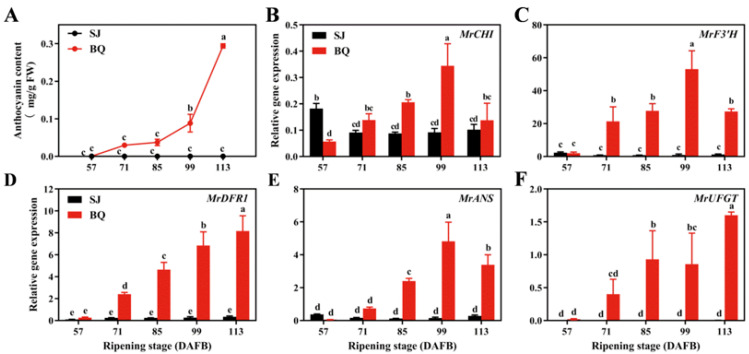
Anthocyanin content (**A**) and relative expression levels of *MrCHI* (**B**), *MrF3′H* (**C**), *MrDFR* (**D**), *MrANS* (**E**), and *MrUFGT* (**F**) involved in anthocyanin pathway in cvs. BQ and SJ during the ripening stage. Error bars represent the SE of the means from three biological replicates. Letters (a, b, c, d and e) indicate samples with statistically significant differences as determined by Duncan’s multiple range test (*p* < 0.05).

During the development of the Chinese bayberry fruit [62], the level of anthocyanin in ‘BQ’ steadily increased, resulting in a distinctive red pigmentation; however, no anthocyanin was observed in ‘SJ’ (Figure 1A). The expression patterns of anthocyanin biosynthetic genes in Chinese bayberry cvs. SJ and BQ were measured using RT-qPCR. The abundance of *MrCHI*, *MrF3′H*, and *MrANS* transcripts in ‘BQ’ tended to increase gradually until 99 DAFB and then decrease, while in ‘SJ’, the expression levels of these genes were lower than in ‘BQ’ throughout the fruit development process (Figure 1B,C,E). Similar to *MrDFR1,* the levels of *MrUFGT* transcript in ‘BQ’ substantially increased up to the end of the ripening stage (Figure 1D,F), while there was little difference in the expression of anthocyanin biosynthetic genes in ‘SJ’ over the sampled days (Figure 1B–F), consistent with the anthocyanin content in Chinese bayberry.

### 3.2. Expression Profiles of MrMYB9 during the Ripening Period and in Specific Tissues

**Figure 2:**
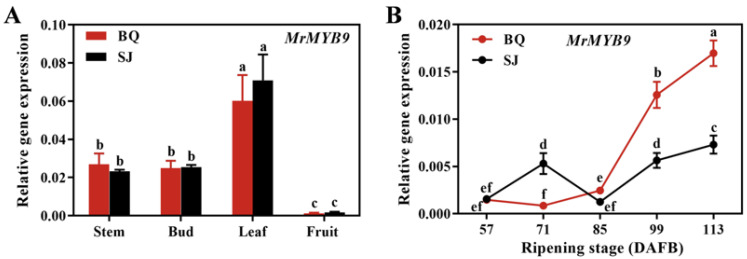
Expression profiles of *MrMYB9* in various tissues (**A**) and during fruit ripening (**B**) of cvs. SJ and BQ. Error bars represent the SE of the means from three biological replicates. Letters (a, b, c, d, e, and f) indicate samples with statistically significant differences as determined by Duncan’s multiple range test (*p* < 0.05).

**Figure 3:**
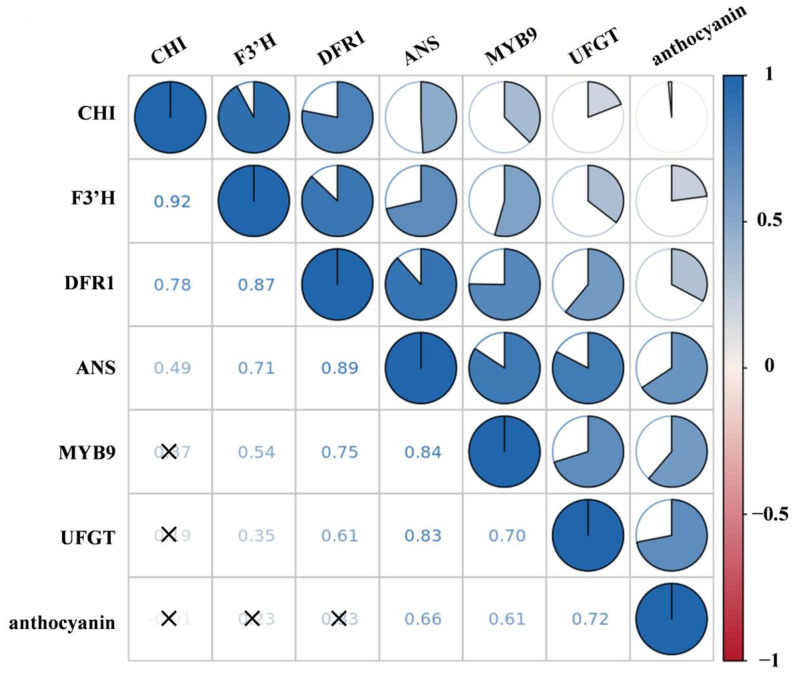
Analysis of correlations between MrMYB9, anthocyanin biosynthesis-related gene expression, and anthocyanin levels during the ripening of cvs. BQ fruits. Blue denotes a positive correlation, while red symbolizes a negative correlation. X denotes non-significant values (*p* > 0.05).

MrMYB9, a putative transcription factor (TF) in the MYB family, was identified from the transcriptome data of Chinese bayberry cvs. SJ and BQ. *MrMYB9* transcript accumulated in all analyzed tissues, including stem, bud, leaf, and fruit, and was highest in the leaf (Figure 2A). During the late maturation stage (99–113 DAFB), the expression level of the *MrMYB9* gene was elevated in both cvs. BQ and SJ fruits, with ‘BQ’ displaying a greater increase compared with ‘SJ’ (Figure 2B). The level of *MrMYB9* expression correlated positively with the anthocyanin content (Figure 3). Positive associations were also observed between the expression level of *MrMYB9* and the anthocyanin metabolic genes, including *MrF3′H*, *MrDFR*, *MrANS*, and *MrUFGT* (Figure 3, R-values in the range 0.54–0.84).

### 3.3. Synteny and Sequence Analysis of MrMYB9 TF

**Figure 4:**
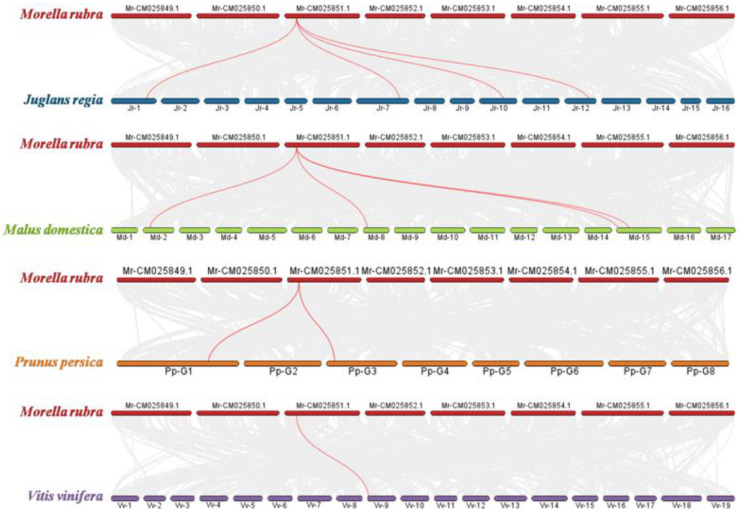
Syntenic analysis of *MYB9* genes between *Morella rubra* and other plant species, including *Juglans regia*, *Prunus persica*, *Malus domestica*, and *Vitis vinifera*. Gray lines in the backgroud show collinear blocks within *Morella rubra* and other plant genomes. The red lines indicate segmentally duplicated *MYB9* gene pairs.

To further elucidate the evolutionary relationship of *MrMYB9*, syntenic maps between *Morica rubra* and *Arabidopsis thaliana*, *Juglans regia*, *Prunus persica*, *Malus domestica*, and *Vitis vinifera* were constructed (Figure 4). Four, four, two, and one homologous *MYB9* gene pairs were discovered between *M. rubra* and *J. regia*, *M. domestica*, *P. persica*, and *V. vinifera*, respectively (Figure 4), but none were identified between *M. rubra* and *A. thaliana* (Figure S1), indicating a closer evolutionary relationship between *M. rubra* and *J. regia*, *M. domestica*, and *P. persica*.

**Figure 5:**
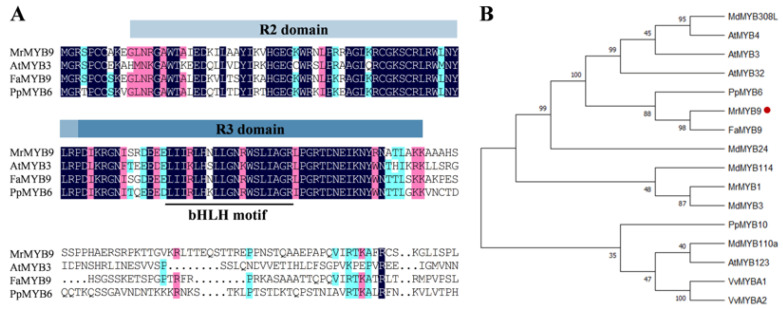
Protein sequence alignment (**A**) and phylogenetic tree (**B**) of MrMYB9 (red dot) and R2R3 MYB activators that modulate the synthesis of anthocyanin in other species. These are the GenBank accession: *Fragaria ananasa* FaMYB9 (OK001453); *Prunus persica* PpMYB6 (XM_007226528); *Morella rubra* MrMYB1 (GQ340767); *Arabidopsis thaliana* AtMYB3 (At1g22640), AtMYB4 (AT4G38620), AtMYB32 (AT4G34990), and AtMYB123 (AT5G35550); *Vitis vinifera* VvMYBA1 (BAD18977) and VvMYBA2 (BAD18978), and *Malus domestica* MdMYB3 (AEX08668), MdMYB23 (AAZ20439), MdMYB110a (BAM84362), and MdMYB308L (MDP0000950559).

The ORF of *MrMYB9* encodes a protein with 303 amino acids. Sequence alignments of MrMYB9 and MYB TFs of other species involved in anthocyanin biosynthesis revealed conserved R2R3 repeats. The presence of a bHLH-binding domain in the R3 repeat region of these proteins (Figure 5A) suggests that they all interact with bHLH TFs via protein–protein interactions. Figure 5B depicts the clustering of MrMYB9 with R2R3-type MYB proteins associated with anthocyanin biosynthesis in other plants based on phylogenetic analysis. *Morella rubra* MrMYB9, *Fragaria ananassa* FaMYB9, and *Prunus persica* PpMYB6 were 55% and 40% identical, respectively.

### 3.4. Subcellular Localization of MrMYB9

**Figure 6:**
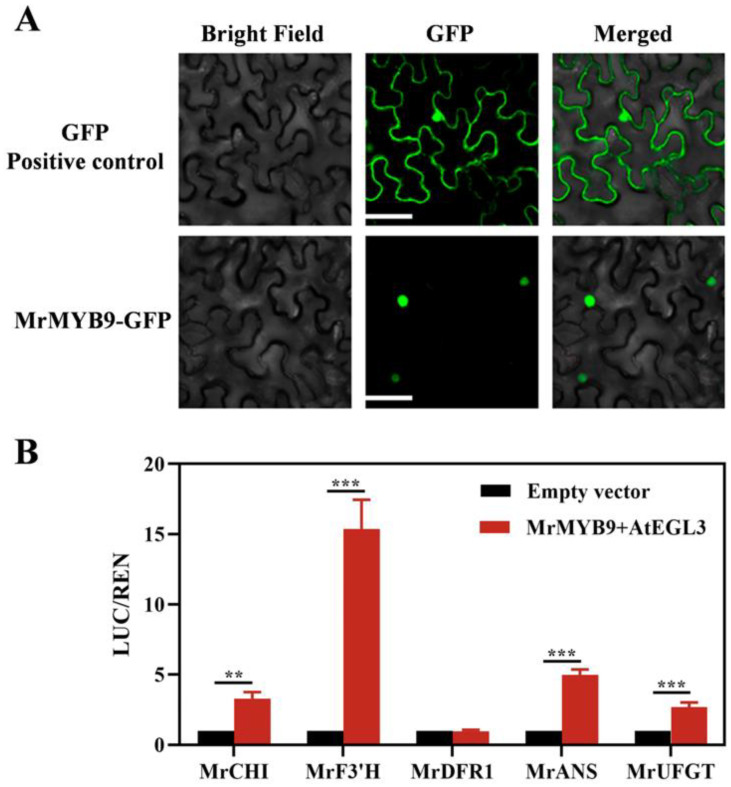
Subcellular localization (**A**) and regulatory effects (**B**) of MrMYB9 on promoters of anthocyanin biosynthesis-related genes in leaf epidermal tissue cells of *Nicotiana benthamiana*. The empty vector plus the promoter’s LUC/REN ratio was set to 1. Asterisks represent significant differences between the empty vector and MrMYB9 (** *p* < 0.01 and *** *p* < 0.001). Scale bar: 50 μm.

For subcellular localization analysis, a MrMYB9–GFP vector expressing the MrMYB9 protein with a GFP tag fused to its C-terminus was constructed. Free GFP was used as a marker to distinguish the nucleus and cytoplasm in the leaves of *N. benthamiana* through agro-infiltration. The MrMYB9–GFP expression system was exclusively localized within the nucleus based on confocal microscopy (Figure 6A).

### 3.5. Regulatory Effect of MrMYB9 on Anthocyanin Biosynthesis-Related Genes

Figure 6B shows that MrMYB9 substantially induced the activities of *MrCHI*, *MrF3’H*, and *MrANS* promoters when co-expressed with the *AtEGL3* gene. Notably, the combination of *MrMYB9* and *AtEGL3* activated the *MrUFGT* promoter in the anthocyanin synthesis branch, whereas *MrMYB9* alone had no effect (Figure S2). These findings suggest that MrMYB9 TF may regulate the anthocyanin pathway during the maturation of Chinese bayberry fruits.

## 4. Discussion

Anthocyanins are natural pigments that are extensively produced in plants and are responsible for giving certain plant organs their characteristic red, purple, and blue colors. In addition to attracting pollinators and seed dispersers [2], they also play crucial roles in protecting plants from biotic and abiotic stressors [2,3,4,5]. Additionally, as a phytonutrient, anthocyanins have high antioxidant and anti-mutant activities and are beneficial to human health [63]. Increasing evidence indicates that anthocyanin ingestion lowers the risk of cardiovascular disease, age-related degenerative diseases, and various kinds of cancers [64,65,66,67].

Several different types of enzymes mediate and control anthocyanin biosynthesis in higher plants. On the one hand, MYB transcription factors bind directly to and modulate the expression of early biosynthetic genes (EBGs) involved in the anthocyanin biosynthesis process, among which are *CHS*, *CHI*, *F3H*, and *F3’H* genes. On the other hand, the MYB–bHLH–WD40 (MBW) ternary protein complex modulates late biosynthesis genes (LBGs), *DFR*, and *UFGT*, which control the downstream accumulation of anthocyanins [68,69]. The major TFs coordinating anthocyanin biosynthesis are members of the R2R3-MYB subfamily, both activators and repressors. However, the MYB TFs associated with anthocyanin biosynthesis in Chinese bayberry have not been well characterized.

Many R2R3-MYB activators have been discovered to be positively associated with the anthocyanin level and the transcripts of anthocyanin biosynthetic genes. During fruit maturation, the red-flesh cortex phenotype of apples was correlated with increased expression of *MYB110a* [70], and apple anthocyanin content was strongly correlated with the expression of *MdMYB10* [30]. Rahim et al. [35] discovered an association between the expression of *MYB10.1* and *MYB10.3* and the expression of *CHS*, *F3H*, and *UFGT* in peach tissues. According to research, the level of *OsMYB3* transcript in black rice was much higher than in white rice, and *OsMYB3* knockout reduced anthocyanin metabolites in grains, indicating that OsMYB3 plays a role in anthocyanin biosynthesis in black rice [71]. A total of seven R2R3-MYB genes had different expression in five species of *Brassica* with purple and green leaves, revealing their association with the biosynthesis of anthocyanin [72]. MrMYB9, an R2R3-MYB transcription factor derived from bayberry, was identified in the current research. Interestingly, the level of *MrMYB9* transcript was low at the initial stage of bayberry development (57 DAFB) and high at the harvest stage (99–113 DAFB) (Figure 2B), paralleling the trend of anthocyanin content and anthocyanin biosynthetic gene expression (Figure 1). Moreover, the positive correlation between *MrMYB9* transcripts and the anthocyanin biosynthetic genes *MrF3′H*, *MrDFR*, *MrANS*, and *MrUFGT* (Figure 3, R-values ranging from 0.54 to 0.84) suggests that MrMYB9 regulates anthocyanin biosynthesis throughout maturation in Chinese bayberry fruit.

Consistent with the Chinese bayberry genome study [57], syntenic maps demonstrated that *M. rubra* has a closer evolutionary relationship with *J. regia*, *M. domestica*, and *P. persica* (Figure 4). Analysis of the protein sequence revealed that the MrMYB9 protein contained a conserved R2R3 repeat, and the presence of a bHLH-binding domain in the R3 repeat segment of these proteins (Figure 5A) suggests a protein–protein interaction with bHLH TFs, as observed in previous research. Phylogenetic analysis positioned MrMY9 within a clade of FaMYB9-like co-activators in anthocyanin and proanthocyanidin pathways (Figure 5B) such as strawberry FaMYB9 [73], apple MdMYB308L [29], peach PpMYB6 [36], *Arabidopsis thaliana* AtMYB3 [74], AtMYB4 [75], and AtMYB32 [74]. Consequently, MrMYB9 exhibits all the structural features of known activators of anthocyanin and proanthocyanidin biosynthesis and may possess comparable regulatory functions. In addition, the localization of the MrMYB9 protein to the nucleus (Figure 6A) supports its potential transcription factor function.

It is well established that co-activators from the bHLH and WD40 repeat families collaborate with R2R3 MYB transcription factors to control the expression of genes involved in anthocyanin biosynthesis. According to research on apples, MdMYB308L interacted with MdbHLH33 and activated the expression of *MdCBF2* and *MdDFR*, functioning as a positive regulator in anthocyanin accumulation [29]. Cui et al. [23] revealed that the transcriptional activation protein complex composed of the transcription factors PyMYB10, PybHLH, and PyWD40 modulated anthocyanin biosynthesis in Yunnan red pear. In the composition of MBW in strawberries, FaMYB5 was an R2R3-MYB activator that positively regulated anthocyanin biosynthesis by trans-activating the *F3’H* [34]. The *Arabidopsis* bHLH protein, which is encoded by the *AtEGL3* gene, is required for MYB transcription factors to activate the promoters of anthocyanidin pathway genes in various plant species [76,77]. According to our study, MrMYB9 greatly increased the activity of the *MrCHI*, *MrF3’H*, and *MrANS* promoters when the *AtEGL3* gene was co-expressed (Figure 6B). Notably, the combination of *MrMYB9* and *AtEGL3* also activated the *MrUFGT* promoter in the anthocyanin synthesis branch (Figure 6B). These findings showed that MrMYB9 acted as an activator of anthocyanin accumulation by trans-activating both EBGs and LBGs, suggesting that the *Morella rubra* MrMYB9 TF functions as a general regulator of the anthocyanin pathway during the development of the Chinese bayberry fruit (Figure S3). It should be noted that MrMYB9 did not activate *MrUFGT* promoters in the anthocyanin-specific pathway in the absence of any partners (Figure S2), indicating that additional research into the regulatory mechanism is required.

## 5. Conclusions

In conclusion, MrMYB9, an R2R3-MYB transcriptional factor found in Chinese bayberry, is likely a positive regulator of anthocyanin biosynthesis. The MrMYB9 transcript was positively correlated with anthocyanin accumulation and the transcript levels of anthocyanin metabolic genes throughout the development of the Chinese bayberry fruit. Furthermore, MrMYB9 significantly transactivated the promoters of anthocyanin biosynthesis-related EBGs (*MrCHI*, *MrF3’H*, and *MrANS*) as well as LBGs (*MrUFGT*) when the *AtEGL3* gene was co-expressed. This finding raises the possibility that MYBs and bHLHs can form complexes that effectively control the expression of their target genes. According to our research, MrMYB9 may actively influence the amount of anthocyanin in Chinese bayberry. However, additional research is necessary to understand the regulatory mechanism underlying MrMYB9-mediated anthocyanin synthesis.

## Data Availability

Data can be provided on suitable request.

## Supplementary Materials

The following supporting information can be downloaded at: https://www.mdpi.com/article/10.3390/biology12070894/s1, Figure S1: Syntenic analysis of *MYB9* gene between *Morella rubra* and *Arabidopsis thaliana*. Gray lines in the background show collinear blocks within *M. rubra* and *A. thaliana*.; Figure S2: Regulatory effects of MrMYB9 alone on promoters of anthocyanin biosynthesis-related genes in leaf epidermal tissue cells of *Nicotiana benthamiana*. The empty vector plus promoter’s LUC/REN ratio was set to 1. Asterisk represents a significant difference between the empty vector and MrMYB9 (** *p* < 0.01 and *** *p* < 0.001).; Figure S3: Schematic of MrMYB9 regulating the anthocyanin pathway of *Myrica rubra*.; Table S1: Primer sequences used for vector construction and RT-qPCR analysis.
