# Supplementary material for: Involvement of a MYB Transcription Factor in Anthocyanin Biosynthesis during Chinese Bayberry (Morella rubra) Fruit Ripening
Source: Biology (Basel). 2023 Jun 21;12(7):894. doi: 10.3390/biology12070894 (PMC10376099; doi:10.3390/biology12070894)
Supplement: Supplementary file 1 [file biology-12-00894-s001.zip › biology-2434331-supplementary figures.pdf]

Supplementary Figures

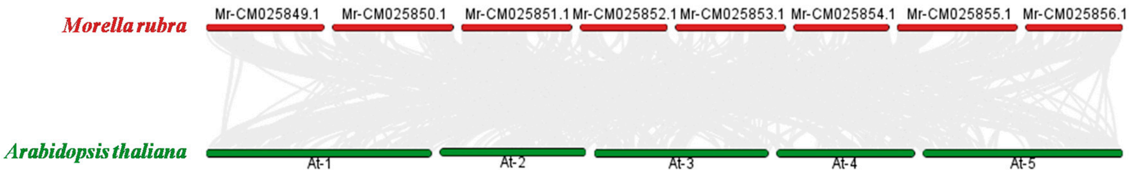

Figure S1. Syntenic analysis of *MYB9* gene between *Morella rubra* and *Arabidopsis thaliana*. Gray lines in the background show collinear blocks within *M. rubra* and *A. thaliana*.

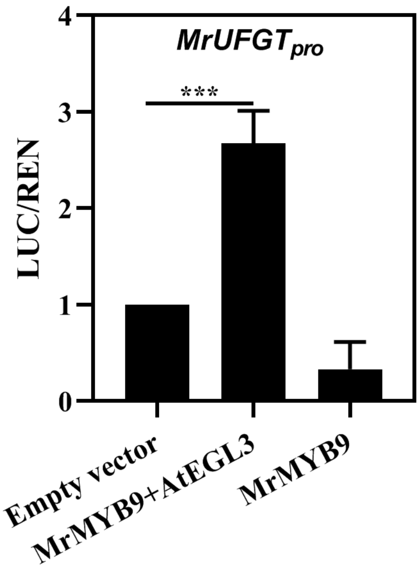

Figure S2. Regulatory effects of MrMYB9 alone on promoters of anthocyanin biosynthesis-related genes in leaf epidermal tissue cells of *Nicotiana benthamiana*. The empty vector plus promoter's LUC/REN ratio was set to 1. Asterisk represents a significant difference between the empty vector and MrMYB9 (*** $p < 0.001$).

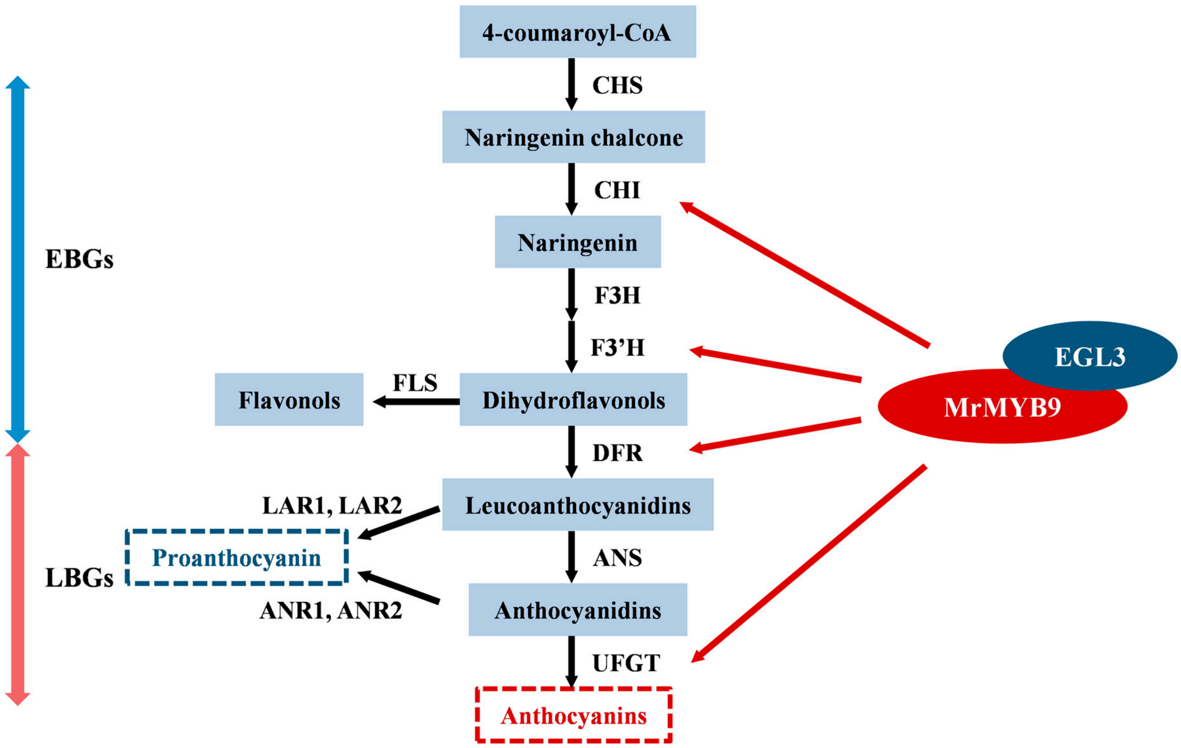

Figure S3. Schematic of MrMYB9 regulating the anthocyanin pathway of *Myrica rubra*.
