# Supplementary material for: Involvement of a MYB Transcription Factor in Anthocyanin Biosynthesis during Chinese Bayberry (Morella rubra) Fruit Ripening
Source: Biology (Basel). 2023 Jun 21;12(7):894. doi: 10.3390/biology12070894 (PMC10376099; doi:10.3390/biology12070894)
Supplement: Supplementary file 1 [file biology-12-00894-s001.zip › Supplementary table 1.pdf]

Table S1. Primer sequences used for vectorconstruction and RT-qPCR analysis

Gene name	Primer sequence (5'-3')	Description
MrMYB9-NF	acgggggactcttgaccatggCCATGGGGAGAAGTCCTTGTT	Primers for pCAMBIA1301-MrMY9-GFP by *Nco* I and *Spe* I
MrMYB9-SR	gcccttgctcaccatactagtTTGCCATTCCTTCGAATCAAG	
MrMYB9-BF	cgctctagaactagtggatccATGGGGAGAAGTCCTTGTTGC	Primers for pGreenII 62-SK-MrMYB9 by *Bam*H I and *Hin*d III
MrMYB9-HR	gtcgacggtatcgataagcttTCATTGCCATTCCTTCGAATC	
AtEGL3-BF	cgctctagaactagtggatccATGGCAACCGGAGAAAACAG	Primers for pGreenII 62-SK-AtEGL3 by *Bam*H I and *Hin*d III
AtEGL3-HR	gtcgacggtatcgataagcttTTAACATATCCATGCAACCCTTT	
MrCHI-BF	ttcctgcagcccgggggatccGCCCAATAAGCAAAGACCTAGC	Primers for pGreenII 0800-MrCHI-pro by *Bam*H I and *Nco* I
MrCHI-NR	tgtttttggcgtcttccatggTCAAGGAGATATATATTTCCTCTGTGC	
MrF3'H-BF	ttcctgcagcccgggggatccAAAAAGTCGGTAAAAC	Primers for pGreenII 0800-MrF3'H-pro by *Bam*H I and *Nco* I
MrF3'H-NR	tgtttttggcgtcttccatggTTTCGGTCCTCGTATG	
MrDFR1-BF	ttcctgcagcccgggggatccATTGAGTTGGTCGGGATG	Primers for pGreenII 0800-DFR1-pro by *Bam*H I and *Nco* I
MrDFR1-NR	tgtttttggcgtcttccatggGCTCCTGCTCCTCAC	
MrANS-BF	ttcctgcagcccgggggatccTCATAGTGTTTGTTTTC	Primers for pGreenII 0800-ANS-pro by *Bam*H I and *Nco* I
MrANS-NR	tgtttttggcgtcttccatggTAATAGCAATATATG	
MrUFGT-BF	ttcctgcagcccgggggatccATTTCGATAAACAACCACCCTC	Primers for pGreenII 0800-UFGT-pro by *Bam*H I and *Nco* I
MrUFGT-NR	tgtttttggcgtcttccatggTTGGACAAAGTTGCTGCAGTTG	
qMrMYB9-F	GCCCAGGACTCCGATTTCTTC	Primers for RT-qPCR
qMrMYB9-R	TCGTAGTTCTCATTCGCACCATC	
qMrActin-F	AATGGAACTGGAATGGTCAAGG	Internal reference gene primers for RT-qPCR
qMrActin-R	CCCGACATAGGCATCTTTCTG	
